# Hemodynamic Response to Upper Airway Obstruction in Hypertensive and Normotensive Pregnant Women

**DOI:** 10.1155/2016/9816494

**Published:** 2016-11-15

**Authors:** John Reid, Riley A. Glew, Joe Mink, John Gjevre, Mark Fenton, Robert Skomro, Femi Olatunbosun

**Affiliations:** ^1^University of British Columbia, Vancouver, BC, Canada; ^2^University of Saskatchewan, Saskatoon, SK, Canada

## Abstract

*Background.* Mild obstructive sleep apnea is common in pregnancy and may have an exacerbating role in gestational hypertension, although currently the interaction between these two diseases is uncertain.* Methods.* We analyzed 43 pregnant subjects, 28 with gestational hypertension (GH) and 15 with normal healthy pregnancy, by level I polysomnography. Additionally, diastolic and systolic blood pressure changes in response to obstructive respiratory events were measured by noninvasive beat-by-beat monitoring. We also assessed a subgroup (*n* = 27) of women with respiratory disturbance indexes <5, for blood pressure responses to very subtle obstructive respiratory disturbances (“airflow reductions”).* Results.* The mean ± standard deviation respiratory disturbance index of our 28 GH women and 15 healthy pregnant women was 10.1 ± 9.9 mmHg and 3.0 ± 3.8 mmHg, respectively. Systolic and diastolic pressure responses to these events were 30.1 ± 12.8 mmHg and 16.0 ± 6.1 mmHg for GH women and 29.1 ± 14.2 mmHg and 14.3 ± 7.7 mmHg for healthy women. For the 27 women in whom we assessed for airflow reduction events, the hemodynamic responses were 27.1 ± 12.3 mmHg systolic and 14.4 ± 6.7 mmHg diastolic.* Interpretation.* Upper airway obstructive events of any severity are associated with a substantial transient blood pressure response in both healthy pregnant and GH women. Whether or not these events have a clinically significant impact on women with GH remains uncertain.

## 1. Introduction

Gestational hypertension (GH) is the commonest medical complication of pregnancy. Obstructive sleep apnea (OSA) is a very common condition and is disproportionately represented in women with GH compared to women with uncomplicated pregnancies [1–4]. OSA is a known risk factor for chronic hypertension and may have a causal role in the development or progression of GH [5]. Transient hypoxemia and/or neuronal activation from obstructive respiratory events could serve as a stimulus for episodic nocturnal rises in blood pressure and vascular reactivity in women with GH [6], theoretically compromising fetal blood flow. Edwards et al. previously demonstrated that the hemodynamic response to obstructive respiratory events was magnified in women with OSA and preeclampsia, when compared to normotensive pregnant women with known OSA [7]. Their explanation was that impaired vascular function due to the preeclampsia was the cause for increased vasoreactive response to an OSA-derived stimulus. Whether or not this heightened response to OSA events leads to worsened clinical outcomes is uncertain. However, the same authors demonstrated, in a small study of 11 women who had GH with proteinuria, that a single night of nasal continuous positive airway pressure (CPAP) improved nocturnal blood pressure and metabolic markers of disease activity [5]. These results are particularly notable because all of the subjects had a respiratory disturbance index (RDI) of <5 events per hour and so by conventional criteria would not be considered to have had OSA.

We have previously shown a high rate of at least mild OSA in women with GH [1]. As part of our study protocol, a subgroup of women had continuous, beat-by-beat noninvasive blood pressure measurements recorded. Using this technology, we assessed the hemodynamic responses to obstructive events in women with GH compared to normal healthy pregnant controls.

Furthermore, we assessed the hemodynamic responses to very mild airway obstruction events, even in subjects who fell below the diagnostic threshold for OSA. To test the hypothesis that even mild upper airway obstruction could have hemodynamically significant consequences, we identified discrete events of very subtle upper airway obstruction—events that had features of inspiratory airflow limitation but did not meet conventional scoring criteria for apnea, hypopnea, or respiratory event related arousal (RERA)—to which we gave the term “airflow reduction.” We then selected those women who had a respiratory disturbance index (RDI) < 5/hr and evaluated the blood pressure responses to these airflow reduction (AR) events in the same manner that we had done for apneas, hypopneas, and RERAs.

## 2. Methods

We previously reported a single centre cross-sectional study which compared women with singleton pregnancies and the diagnosis of GH (with or without proteinuria) to healthy women with uncomplicated singleton pregnancies of similar gestational age [1]. As a substudy, we evaluated the hemodynamic responses to obstructive respiratory events in subjects who wore the Portapres® device for the study night.

### 2.1. Participants

Detailed methods of the original study have been previously published [1]. In short, women ≥18 years of age with singleton pregnancies and the diagnosis of GH (with or without proteinuria) were recruited from the Fetal Assessment Unit and Antepartum Ward of Royal University Hospital, Saskatoon, Saskatchewan, Canada, between February 2006 and February 2008. GH was defined according to standard criteria [8]. Exclusion criteria included multiparity; imminent delivery; severe underlying maternal or fetal conditions expected to worsen maternal or fetal outcomes; and maternal condition preventing safe transfer off the obstetrical floor. Women with chronic hypertension were considered eligible, providing they met the criteria of chronic hypertension with superimposed GH [8]. Healthy pregnant subjects were recruited by local advertising.

Written informed consent was obtained for all patients included in the study protocol, which was approved by the University of Saskatchewan Biomedical Ethics Review Board and registered with the National Institute of Health Clinical Trials Registry (Identifier NCT00259688).

### 2.2. Procedures

All subjects underwent full night in-lab level I polysomnography (PSG) (Sandman 8.0, Tyco Inc., Ottawa, Canada) evaluation. As per the American Academy of Sleep Medicine (AASM) criteria [9], respiratory events were categorized as apneas (a decrease in airflow by ≥90% from baseline for at least 10 seconds); hypopneas (decrease in airflow by ≥30% for at least 10 seconds, followed by a desaturation of ≥4% from the preevent baseline); and RERAs (a sequence of breaths lasting at least 10 seconds, associated with flattening of the nasal pressure waveform leading to an arousal from sleep, and the sequence does not meet criteria for an apnea or hypopnea). All scoring was performed by a single registered PSG technician, who was blinded to GH status and blood pressure measurements. The total number of apneas, hypopneas, and RERAs divided by the hours of sleep was expressed as the RDI.

In addition to standard PSG evaluation described above, we also monitored continuous blood pressure via finger arterial waveform analysis with a Portapres unit (Finapres Medical Systems, Amsterdam, Netherlands). This system allows for noninvasive, beat-to-beat analysis of blood pressure, which controls for the height of the monitor in relation to the heart. It has been validated against brachial sphygmomanometer measurements in healthy and hypertensive pregnant women, meeting the Association for the Advancement of Medical Instrumentation criteria for acceptability, and has achieved a British Hypertension Society grading of “B” for diastolic blood pressure and “C” for systolic blood pressure measurement in pregnancy [10]. Blood pressure measurements were recorded directly into the Sandman software so they could be assessed in temporal relation to other PSG events.

Blood pressure and heart rate responses to obstructive respiratory events were assessed using locally designed, custom software, with target measurements patterned from previously published work [7]. We collected hemodynamic data 10 seconds prior to obstructive respiratory events and 15 seconds after event termination. For systolic, diastolic, and mean blood pressure, we measured the difference between preevent minimum and postevent maximum measurements. The software also considered events which occurred in rapid eye movement (REM) and non-REM (NREM) sleep as well as those which were and were not associated with an arterial oxygen desaturation of ≥3%. The program was designed to screen out “bad” data as per a predetermined algorithm. The data was then screened by a single investigator (JM, the software developer who was blind to all other subject data) to ensure that it was accurate. The hemodynamic response was then calculated as the difference in the mean pre- and postobstructive respiratory event hemodynamic measures.

**Figure 1:**
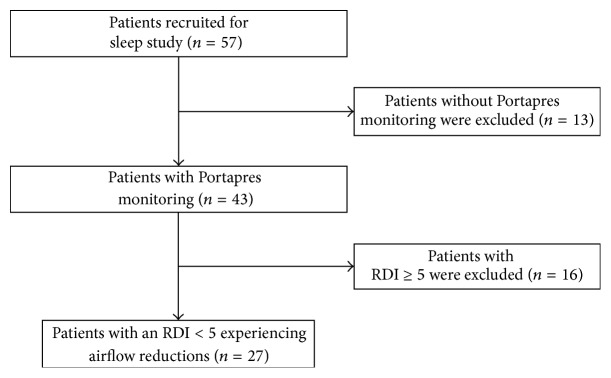
Study population flow chart. Among the 43 patients with Portapres monitoring, 4 patients had no apnea, hypopnea, or RERA (respiratory event related arousal) events, but these patients are included among the 27 patients with an RDI < 5.

Due to expense, we had only one Portapres device but otherwise had capacity to study two subjects per night by PSG (which we did whenever possible). If GH and healthy woman were being studied on the same night, the Portapres was used for the GH woman. Otherwise, the subject who wore the Portapres was chosen by random draw. Additionally, in some subjects, the device did not record consistently well throughout substantial portions of the night. Again, all data was manually reviewed by a single investigatory (JM), to ensure a reasonable time of acceptable quality data collection. A total of 43 subjects were ultimately included in our analysis. A summary of the study population is provided in Figure 1.

We compared the hemodynamic responses to obstructive respiratory events (apnea, hypopnea, and RERAs) in the GH versus healthy pregnant groups. In an additional analysis, we assessed only the women whose RDI < 5 for AR events. Scoring of AR events was performed by a single investigator (JKR) who was blinded to patient identifying data, GH status, and blood pressure recording. We defined AR as a sequence of breaths lasting at least 10 seconds characterized by increasing respiratory effort or flattening of the nasal pressure waveform (this part is the AASM definition RERA [9]) followed by evidence of apparent physiological perturbation (increase in chin EMG signal, leg kicking, increase in frequency of EEG or series of K-complexes, or obvious increase in pulse rate) that does not meet the AASM criteria for an arousal and can therefore not be scored as RERA. Hemodynamic responses to AR events in this subgroup of women were assessed in the same manner as that for apneas, hypopneas, and RERAs.

### 2.3. Statistical Analysis

Comparisons of the patient characteristics were made between subjects with healthy pregnancies and those with GH. Normality was assessed with the Shapiro-Wilk test, QQ plots, and histograms. Means were compared for normally distributed variables, while the distributions of variables that were not normally distributed were compared with the Mann–Whitney *U* test. Categorical variables consisting of count data were compared using *χ* ^2^ test.

Blood pressure response to obstructive respiratory events was measured as the difference between postevent blood pressure and minimum preevent blood pressure. Results are presented in tables and figures as the means of these min-max differences. Hemodynamic responses were compared according to the type of sleep disturbance, sleep stage, pregnancy status, oxygen desaturation, and diagnosed sleep disordered breathing. Normality of the hemodynamic variables was determined as previously described. The distributions of nonnormal variables were compared with the Friedman test (heart rate and oxygen saturation). Comparisons of normally distributed variables used a generalized linear model to estimate adjusted means (systolic and diastolic blood pressure). Generalized estimating equations were used to account for repeated measures, using a working correlation matrix to estimate robust standard errors. The same analytic steps were implemented for the AR data. Data was recorded and stored with Microsoft Excel, with all data processing and analysis conducted with SAS software version 9.3 (SAS Institute Inc., Cary, NC, USA). Statistical significance was assessed using *α* = 0.05.

## 3. Results

A total of 43 subjects were included in the analysis, 28 with GH and 15 with uncomplicated pregnancies. Baseline characteristics are presented in Table 1. Not surprisingly, women in the GH group had a higher BMI, were more likely to report snoring, and had less total sleep time, less REM sleep, and lower sleep efficiency on PSG (Table 1). The GH group also had a higher RDI (Table 1), consistent with the findings of the parent study [1] from which this analysis was derived. In addition, the mean nocturnal blood pressure was clearly higher in the GH group than in controls (Table 1).

We initially analyzed the hemodynamic responses to conventionally scored apneas, hypopneas, and RERAs in the 43 subjects. Four of those subjects did not have any events and therefore had no data to analyze. Of the remaining 39 subjects, a total of 889 events were available for analysis (Table 2). We found that all measured events resulted in a substantial blood pressure response (Table 2). Blood pressure increased by a mean ± standard deviation (SD) of 30.0 ± 13.0 mmHg systolic and 15.7 ± 6.4 mmHg diastolic (Table 2). This response was quite consistent regardless of GH versus healthy status, stage of sleep, type of event (apnea/hypopnea, RERA), diagnosed sleep disordered breathing (RDI ≥ 5 versus RDI < 5), or whether or not there was an associated oxygen desaturation (Table 2).

**Figure 2:**
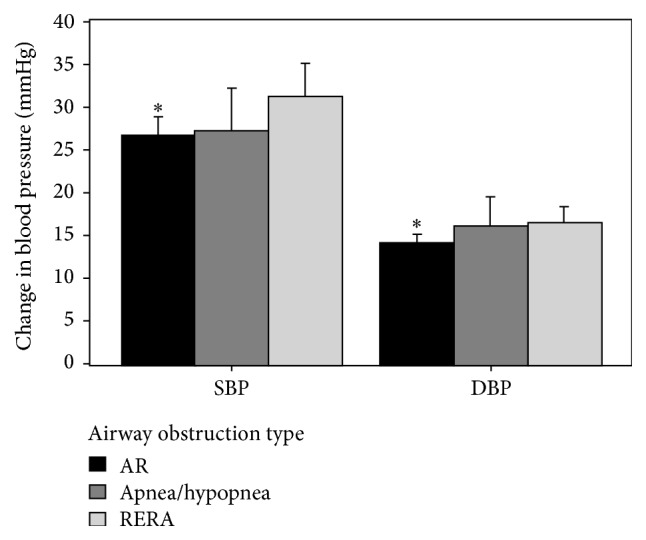
Blood pressure responses to RERA, apnea/hypopnea, and AR events among patients (*n* = 27) with RDI < 5. Data are the modeled response (mean ± 95% confidence interval) adjusted for repeated measures. AR, airflow reduction; RERA, respiratory event related arousal; mmHg, millimeters of mercury; ^*∗*^ *p* < 0.05.

We then analyzed only the women with RDI < 5 (*n* = 27) for AR events. In this subgroup ARs were much more common than apneas/hypopneas (*n* = 20) or RERAs (*n* = 194), with 1606 AR events in total for 27 subjects (Table 3), yielding an “airflow reduction index” (ARI) (mean ± SD) of 12.0 ± 1.4 events per hour (Table 3). For comparison, the RDI (mean ± SD) of this subgroup of patients was 2.3 ± 1.6. The ARI for the GH and healthy patients and between REM and NREM sleep were equal (Table 3). The hemodynamic responses among this group of patients for all events showed an increase (mean ± SD) of 27.1 ± 12.3 mmHg systolic and 14.4 ± 6.7 mmHg diastolic. The largest blood pressure responses (systolic: 31.2 ± 13.7 mmHg, diastolic: 16.5 ± 7.5 mmHg) were observed for RERAs (Figure 2). No difference (*p* > 0.05) in hemodynamic response was identified between apnea/hypopnea versus RERA events. Compared to RERA related events, both systolic (26.6 ± 12.1 mmHg, *p* = 0.01) and diastolic (14.2 ± 6.6 mmHg, *p* = 0.003) blood pressure responses were significantly lower for AR events but still represented impressive hemodynamic perturbations.

## 4. Discussion

Contrary to the findings of Edwards et al. [7], we did not find a significant difference in blood pressure responses of GH versus healthy pregnant women to obstructive respiratory events. There are a few potential explanations for this discrepancy. Firstly, Edwards studied a much smaller and homogenous group of women with severe preeclampsia [7]. Our study was more ambitious in terms of numbers of subjects enrolled, but our subjects were more heterogeneous, many of whom did not have proteinuria. The diurnal blood pressure control and inherent vasoreactivity will vary somewhat between healthy pregnancy, GH with or without preexisting hypertension, and GH with proteinuria. While our GH subjects clearly had higher mean nocturnal blood pressure compared to the healthy controls (Table 1), many had reasonably well controlled hypertension at the time of study. Furthermore, most of our subjects either did not have frank OSA or had much milder OSA than reported by Edwards' study [7]. Milder sleep disordered breathing as well as milder and/or better controlled GH may have resulted in us not detecting a difference between our study group and healthy controls.

Nonetheless we did find that obstructive respiratory events resulted in a substantial blood pressure response for all women in our study. It is impressive that the hemodynamic response was comparable across all levels of severity of airway obstructive events, even in the AR subgroup, for whom the RDI was <5. We think this finding is particularly provocative because of the much higher frequency of subtle upper airway obstructive events observed (ARI of 12.0 ± 1.4 versus RDI of 2.3 ± 1.6 in this study subset), suggesting that even these women who are considered “normal” by conventional scoring methods may still have a high burden of inspiratory airflow limitation during sleep, with potential physiologic consequences. In fact, given the much greater frequency of ARs than conventionally scored events, it could be postulated that they impose a much greater hemodynamic consequence on the patient. The clinical significance of this finding is uncertain but it is congruent with Edwards et al.'s finding that the application of nasal CPAP improved blood pressure in preeclamptic women who had increased inspiratory airflow resistance but did not have OSA [5].

It is important to note that the hemodynamic responses we observed in GH women to upper airway obstructive events were equally matched by the responses seen in healthy pregnant women. It could be reasonably argued then that these brief responses are inconsequential. While that remains a potential explanation, when our findings are taken in the context of Edwards et al.'s findings of improved blood pressure increments with nocturnal CPAP in preeclamptic women [5], it begs the question as to whether the physiologic consequences of these hemodynamic perturbations depend on the underlying vascular reactivity of the patient. Perhaps these events are without consequence in healthy pregnant women or those with mild hypertension but are important in women who already have increased vasoreactivity and compromised fetal blood flow. This is an interesting and important question, but one that cannot be answered currently by the literature.

Another questioned raised by this study is whether or not the conventional scoring criteria for OSA, which was developed in nonpregnant subjects (mostly middle-aged men), is appropriate for use in pregnant patients. Pregnancy is associated with a hormonally induced increase in respiratory drive, leading to a mild respiratory alkalosis. This may be somewhat protective against hypoxemia, the major and most objective criterion that allows for obstructive respiratory events to be scored. Consequentially, pregnant women with mild OSA may be set up to be underscored by conventional scoring criteria. At the same time, pregnant women with GH may be physiologically primed to experience greater hemodynamic perturbations with OSA events. A greater awareness of how sleep disordered breathing presents in pregnant women and the hemodynamic consequences thereof is critical if we are to understand the potential role CPAP treatment may have to play in managing GH.

Our study has a number of limitations. Firstly, for practical reasons we were unable to study all of our subjects with the Portapres. Nevertheless, 43 is an impressive number of subjects for this type of research and our study is the largest published to date with this level of monitoring in pregnant and GH women. Additionally, the AR events that we report are not a standardized or validated score. We are, however, not proposing that ARs should be adopted as new scoring criteria. Rather, our intention is only to explore the concept that the mildest of airflow obstructions can result in important physiologic disturbance. That is the reason that we chose to evaluate only those women with RDI < 5, so that we were assessing the subtlest events in the mildest (technically “normal”) subjects. Airflow reductions are not a standardized or validated measurement. The reason we chose only to assess those women with RDI < 5 is to open the question as to whether even these women who are the mildest of the mild still have inspiratory airflow abnormalities. As we have no frame of reference for comparison, we did not test the entire group. We still think, however, that the findings raise an important question that deserves future study.

## 5. Conclusion

There is a substantial transient rise in blood pressure following even very mild upper airway obstructive events in women with both GH and healthy pregnancies. Although we did not find a significant difference between the responses of GH versus healthy women, we did find that the hemodynamic response to even marginal obstructive events (i.e., ARs) is nearly as profound as for conventionally scored events. Furthermore, because these AR events were much more frequent than conventionally scored apneas, hypopneas, or RERAs, they could represent an important physiologic stress in women with a heightened inflammatory and vasoreactive state such as GH. Although provocative, our findings should be considered “pilot” data. We recommend that further research into the interaction of OSA and GH consider all indications of upper airway obstruction as potentially important and not just focus on traditionally scored apneas, hypopneas, and RERAs.

## Figures and Tables

**Table 1:** Characteristics of patients in the study sample with Portapres monitoring. All values are mean ± standard deviation unless otherwise noted.

Patient characteristics	Overall (*n* = 43)	Healthy (*n* = 15)	Gestational hypertension (*n* = 28)	*p* value
Age (years)	29.7 ± 4.7	30.1 ± 3.5	29.4 ± 5.3	0.68^a^
Gestational age (weeks)	34.3 ± 3.2	34.5 ± 3.2	34.2 ± 3.3	0.94^b^
Comorbidities				
BMI (kg/m^2^)	33.9 ± 7.6	28.2 ± 3.9	36.9 ± 7.3	<0.0001^a^
Hypertension (yes versus no)	7% (3/40)	0% (0/15)	11% (3/25)	0.19^c^
Proteinuria (yes versus no)	42% (18/25)	0% (0/15)	64% (18/10)	<0.0001^c^
Medications (≥1 versus 0)				
Antihypertensives^*∗*^	51% (22/21)	0% (0/15)	79% (22/6)	<0.0001^c^
Sleeping agents^†^	12% (5/37)	0% (0/15)	20% (5/22)	0.14^c^
Hemodynamics (baseline)^Ψ^				
SBP (mm Hg)	142 ± 26.2	115 ± 26.0	147 ± 22.6	0.0003^a^
DBP (mm Hg)	77.9 ± 17.2	64.6 ± 16.0	80.5 ± 16.2	0.0065^a^
Heart rate (bpm)	76.9 ± 10.5	81.7 ± 8.48	75.9 ± 10.6	0.0488^a^
Oxygen saturation (%)	95.2 ± 1.89	95.0 ± 1.68	95.2 ± 1.92	0.6184^a^
Sleep characteristics				
ESS	8.1 ± 3.7	8.8 ± 4.0	7.7 ± 3.6	0.14^b^
Snoring (yes versus no)	67% (29/14)	40% (6/9)	82% (23/5)	0.008^c^
TST (min)	280 ± 66	317 ± 43	261 ± 69	0.006^a^
Sleep efficiency (%)	67.2 ± 15.2	73.2 ± 8.4	64.0 ± 17.2	0.39^a^
% supine (%)	21.8 ± 27.0	20.9 ± 24.6	22.2 ± 28.7	0.46^b^
% REM (%)	10.2 ± 5.7	13.5 ± 5.4	8.5 ± 5.2	0.004^b^
AHI (events/h)	1.5 ± 2.9	0.6 ± 1.6	2.0 ± 3.3	0.11^b^
RDI (events/h)	7.6 ± 8.9	3.0 ± 3.8	10.1 ± 9.9	0.002^b^
AI (events/h)	13.8 ± 9.0	12.1 ± 7.4	14.8 ± 9.7	0.49^b^

^a^ *t*-test comparison of means. ^b^Mann–Whitney *U* test. ^c^Chi-square test. BMI, body mass index; SBP, systolic blood pressure; DBP, diastolic blood pressure; ESS, Epworth sleepiness scale; REM, rapid eye movement; AHI, apnea hypopneas index; RDI, respiratory disturbance index; AI, apnea index. ^*∗*^Antihypertensive medications included labetalol, nifedipine, and methyldopa. ^†^One participant was missing medication data. All values pertaining to sleep medications are calculated from *n* = 42. ^Ψ^Baseline hemodynamics were calculated from the values recorded *immediately prior* to an apnea, hypopnea, and RERA during Portapres monitoring.

**Table 2:** Hemodynamic responses to apnea, hypopnea, or RERA breathing events.

	*n*_*i*_ (*n*_*ij*_)^a^	ΔSBP (mmHg)	ΔDBP (mmHg)	ΔHeart rate (BPM)	ΔO_2_ saturation (%)
Overall, mean ± SD	39 (889)	30.0 ± 13.0	15.7 ± 6.4	6.6 ± 4.4	1.7 ± 1.9
Event type					
Apnea/hypopnea	17 (157)	28.7 ± 13.0	16.7 ± 7.3	6.7 ± 5.2	4.6 ± 2.6^*∗*^
RERA	39 (732)	30.2 ± 13.0	15.5 ± 6.2	6.6 ± 4.2	1.0 ± 0.9
Sleep stage					
1 to 4 (non-REM)	39 (792)	29.8 ± 12.8	15.5 ± 6.1	6.4 ± 4.1	1.4 ± 1.7^*∗*^
5 (REM)	20 (97)	30.5 ± 15.2	17.6 ± 8.4	8.1 ± 6.3	3.4 ± 2.5
Pregnancy status					
Healthy	26 (148)	29.1 ± 14.2	14.3 ± 7.7	8.5 ± 5.7	1.5 ± 1.3
GH	13 (741)	30.1 ± 12.8	16.0 ± 6.1	6.2 ± 4.0	1.7 ± 2.0
Desaturation					
No (<3%)	39 (713)	29.9 ± 13.1	15.3 ± 6.1	6.5 ± 4.2	0.9 ± 0.7^*∗*^
Yes (≥3%)	21 (176)	29.9 ± 12.9	17.4 ± 7.5	7.0 ± 5.2	4.6 ± 2.3
Sleep disordered breathing					
No (RDI < 5)	23 (214)	30.8 ± 13.4	16.4 ± 7.5	7.9 ± 5.1	1.5 ± 1.6
Yes (RDI ≥ 5)	16 (675)	29.6 ± 12.9	15.5 ± 6.1	6.2 ± 4.1	1.7 ± 2.0

^a^The total number of individuals with hemodynamic measures is 39, which is 4 less than the total sample size (Table 1). There were 4 individuals with no apnea, hypopnea, or RERA events and therefore contributed no hemodynamic data. *n*_*i*_, number of patients; *n*_*ij*_, number of hemodynamic events; SBP, systolic blood pressure; DBP, diastolic blood pressure; mmHg, millimeters of mercury; BPM, beats per minute; RERA, respiratory event related arousal; REM, rapid eye movement; GH, gestational hypertension; SD, standard deviation; ^*∗*^ *p* < 0.0001.

**Table 3:** Airflow reduction index.

	Airflow reduction events (count)	Total sleep time (minutes)	Airflow reduction index (events/hr ± SD)	*p* value^a^
Overall	1606	8044	12.0 ± 1.3	
Sleep stage				
NREM	1410	7158.3	11.8 ± 1.4	0.37
REM	196	885.7	13.3 ± 1.5	
Pregnancy status				
Healthy	815	4139.3	11.8 ± 1.1	0.68
GH	791	3904.7	12.2 ± 1.6	

^a^ *t*-test comparison of means. GH, gestational hypertension; NREM, nonrapid eye movement; REM, rapid eye movement; SD, standard deviation.
